# Adsorption of Fibrinogen on Silica Surfaces—The Effect of Attached Nanoparticles

**DOI:** 10.3390/biom10030413

**Published:** 2020-03-06

**Authors:** Kristin Hyltegren, Mats Hulander, Martin Andersson, Marie Skepö

**Affiliations:** 1 Division of Theoretical Chemistry, Lund University, POB 124, SE-221 00 Lund, Sweden; 2 Applied Chemistry, Chemistry and Chemical Engineering, Chalmers University of Technology, Chalmersplatsen 4, SE-412 96 Göteborg, Sweden; mats.hulander@chalmers.se (M.H.); martin.andersson@chalmers.se (M.A.); 3 LINXS—Lund Institute of Advanced Neutron and X-ray Science, Scheelevägen 19, SE-223 70 Lund, Sweden

**Keywords:** protein adsorption, coarse-grained modeling, fibrinogen, nanoparticles, nanotopography

## Abstract

When a biomaterial is inserted into the body, proteins rapidly adsorb onto its surface, creating a conditioning protein film that functions as a link between the implant and adhering cells. Depending on the nano-roughness of the surface, proteins will adsorb in different amounts, with different conformations and orientations, possibly affecting the subsequent attachment of cells to the surface. Thus, modifications of the surface nanotopography of an implant may prevent biomaterial-associated infections. Fibrinogen is of particular importance since it contains adhesion epitopes that are recognized by both eukaryotic and prokaryotic cells, and can therefore influence the adhesion of bacteria. The aim of this study was to model adsorption of fibrinogen to smooth or nanostructured silica surfaces in an attempt to further understand how surface nanotopography may affect the orientation of the adsorbed fibrinogen molecule. We used a coarse-grained model, where the main body of fibrinogen (visible in the crystal structure) was modeled as rigid and the flexible αC-chains (not visible in the crystal structure) were modeled as completely disordered. We found that the elongated fibrinogen molecule preferably adsorbs in such a way that it protrudes further into solution on a nanostructured surface compared to a flat one. This implicates that the orientation on the flat surface increases its bio-availability.

## 1. Introduction

When a biomaterial is inserted into the body, a protein film consisting of plasma proteins from the blood is immediately formed on the implant surface. The 340 kDa glycoprotein fibrinogen is an abundant protein in the blood of vertebrates and is primarily involved in blood clotting and readily adsorbs to biomaterial surfaces upon implantation. In a wound, thrombin polymerizes fibrinogen into fibrin that, together with platelets, form a clot over the wound. When adsorbed to the surface of a biomaterial, fibrinogen can trigger inflammatory responses and subsequent formation of a fibrous capsule that can lead to failure or loss of function of the biomaterial. The fibrinogen molecule harbors epitopes that are recognized by both human and bacterial cells through specific integrins [1,2,3,4]. An infection on an implant can have severe consequences and is one of the most common causes of implant failure and need for revision surgery. Surface modifications at the nanoscale have previously been suggested as a promising approach to prevent adhesion of bacteria and development of biomaterial-associated infections on implants [5]. A leading cause for biomaterial-associated infections is the bacterium *Staphylococcus epidermidis* [6], which is normally present on human skin. A recent study found that the ability of *S. epidermidis* to adhere on adsorbed fibrinogen is highly dependent on the nanostructure of the underlying substrate, possibly by affecting the orientation or conformation of the adsorbed fibrinogen molecule [7].

In the present study, we used coarse-grained Monte Carlo simulations to investigate how fibrinogen adsorbs to smooth and nanostructured surfaces (with attached nanoparticles). This enabled us to analyze in more detail how fibrinogen adsorbs and explain the experimental findings described above. We investigated the effect of surface charge, the effect of nanoparticle size, the angles between bound fibrinogen and the surface, and the effect of the disordered fragments (the αC-chains) of fibrinogen.

Human fibrinogen consists of two symmetrical halves that each contain three polypeptide chains called Aα (610 amino acid residues), Bβ (461 amino acid residues), and γ (411 amino acid residues). The chains are connected to each other by several disulfide bonds. Fibrinogen folds into a 45-nm-long rod with two thicker nodules (the D domains) at the ends, and one nodule in the middle of the rod (the E domain). The C-terminal 410 amino acid residues of the Aα-chains are not visible in the crystal structure [8] and are here counted as the disordered αC-chains, which extend out from the two D domains (sometimes only the C-terminal 400 residues are included). NMR studies have shown that the αC-regions of bovine fibrinogen each contain a structured domain of approximately 60 amino acid residues [9]. However, the stability of this structure is low. Human fibrinogen seems to have αC-domains with similar structure but even less stable than the bovine ones [10].

**Figure 1:**
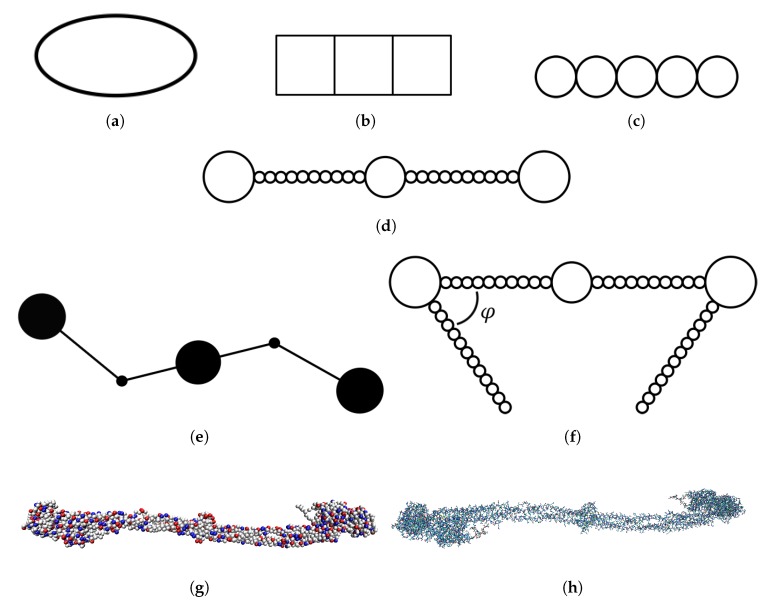
Depiction of models that have been used for simulations of fibrinogen with increasing level of detail: (**a**) ellipsoid model [11]; (**b**) rigid connected squares model [12]; (**c**) rigid linear pentamer model [13]; (**d**) rigid linear chain model [14,15]; (**e**) coarse-grained model with two hinges [17]; (**f**) rigid linear chain model with two side arms and different ϕ angles [16]; (**g**) coarse-grained model from crystal structure, either completely rigid (this work [20]) or somewhat flexible [19]; and (**h**) atomistic model from crystal structure [17].

In one of the simplest models that has been used for simulations of fibrinogen, the protein is approximated as an elongated ellipsoid [11] (see Figure 1a). In another study of fibrinogen adsorption, the protein was described as three connected squares [12] (see Figure 1b). Zhdanov et al. described fibrinogen as a linear pentamer with a monomer diameter of 7.5 nm [13] (see Figure 1c). A somewhat more detailed model that has been used to describe fibrinogen in simulations is a linear chain consisting of 23 touching spheres of different diameters, mimicking the differences in thickness at different parts of the fibrinogen rod [14,15] (see Figure 1d). The spheres at the ends had a diameter of 6.7 nm, the one in the middle 5.3 nm, and the remaining ones 1.5 nm. This model was later extended by adding two side arms representing the αC-chains [16] (see Figure 1f). The side arms were also linear but the angle ϕ between the side arms and the body of the protein was varied.

Atomistic molecular dynamics simulations of the main body of fibrinogen (excluding the αC-chains and other flexible parts not visible in the crystal structure) in solution have also been performed, revealing bending motions of the protein [17]. An atomistic representation of the fibrinogen crystal structure is shown in Figure 1h. The simulations showed that two hinges are responsible for the flexibility of the protein while the rest of the fibrinogen main body does not undergo large conformational changes. Based on these results, a simplified coarse-grained model of fibrinogen was developed (see Figure 1e). In that model, fibrinogen is represented by a stiff central rod connected to two other rods that can pivot around the hinges. The ends of the protein and the central domain are represented as spheres.

The atomistic simulations of fibrinogen in solution were followed by a study of fibrinogen adsorption on mica and graphite, where one half of the symmetric fibrinogen rod was modeled atomistically [18]. The binding of fibrinogen to gold nanoparticles has been studied using a coarse-grained model where fibrinogen was modeled from the crystal structure determined by Kollman et al. [8] and each amino acid residue was represented as a sphere (see Figure 1g). The amino acids interacted via a bonded potential (a sum of potentials for bonds, angles and dihedrals) and a nonbonded potential (two Lennard-Jones-type potentials—one local and one nonlocal) [19].

We used the model depicted in Figure 1g, where each amino acid of the crystal structure is coarse-grained into a sphere. However, we considered the molecule as completely rigid and thus the spheres did not move relative to each other. Such a model was also used by Lopez and Lobaskin [20]. This model has an intermediate level of detail and takes the charge distribution over the molecule into account. This enables us to study the electrostatic effects behind the adsorption of fibrinogen.

We found that the main body of fibrinogen protrudes more into solution the larger the curvature of the surface. The fibrinogen main body is anchored to the surface by one of its D domains in the same way regardless of the curvature, thus the effect on the protein orientation seems to be solely a function of the curvature. Our model included only electrostatic interactions (and excluded volume), but the results are still similar to those of Lopez and Lobaskin, who studied fibrinogen adsorption onto hydrophobic surfaces [20].

It has been suggested that fibrinogen may adsorb to negatively charged surfaces by means of electrostatic interactions between the net positively charged αC-domains and the surface [21,22,23,24]. However, in our present study, the αC-chain was found to have a net negative charge. This difference is likely to be due to the treatment of the histidines. While we treated them as having zero charge, Doolittle et al., for example, used a histidine p$K_{a}$ of 6.7 to calculate the charge of the Aα-chain at pH 7.3, giving a non-negligible charge on the histidines [25].) Our comparison of the free energies of adsorption of the main body and the disordered fragments reveals that there is a stronger attraction between one disordered fragment and the surface than between the fibrinogen main body and the surface.

## 2. Materials and Methods

### 2.1. Coarse-Grained Model

The main body of fibrinogen was modeled as a rigid molecule with a structure equal to the crystal structure of human fibrinogen [8]. There is a slight difference between the two molecules that form the asymmetric unit of the structure, and in this study biological assembly 1 was chosen. Each amino acid was modeled as a sphere with a radius of 2.5 Å. This radius gives the sphere a smaller volume than that of most amino acid residues but a more realistic contact separation between charges, which were modeled as point charges located in the centers of the spheres. The pH was set to 7.4. Therefore, the aspartic acid and glutamic acid residues were fully deprotonated (charge −1) while the lysine and arginine residues were fully protonated (charge +1). The crystal structure also contained four bound calcium ions with a charge of +2 each. The N- and C-terminals were modeled as separate spheres with charges of +1 and −1, respectively. The number of charged histidine residues was estimated to be negligible. All of the positions of the spheres were fixed relative to each other. The synthetic peptides that were present when growing the crystals to determine the crystal structure were not included in our protein model. Since the C-terminal parts of the α-chains were not included in the crystal structure, the charges of the C-terminals of the parts of the α-chains present in the crystal structure were set to zero.

Since some parts of fibrinogen are flexible, most notably the αC-chains, 35% of the amino acid residues were not visible in the electron density maps, and thus they are not part of the crystal structure determined by Kollman et al. [8]. To determine the role of the αC-chains for adsorption we studied an αC-chain (residues 201–610 of the Aα-chain were here counted as the αC-chain since they were not part of the crystal structure) separately using a coarse-grained model that has been used previously for flexible proteins [26,27,28,29,30], with the exception that here the spheres representing amino acids are hard instead of soft. In the present study, the electrostatic interactions were described using Debye–Hückel theory, taking particle size into account using the following expression for the potential energy:

(1) $$w_{ij}=\left\{\begin{array}{cc} \frac{e^{2}}{4\pi \varepsilon_{0}\varepsilon_{r}}\frac{z_{i}z_{j}}{r_{ij}\left(1+\kappa \sigma_{ij}\right)}e^{-\kappa \left(r_{ij}-\sigma_{ij}\right)}, & r_{ij}\geq \sigma_{ij}\\ \infty , & r_{ij}<\sigma_{ij} \end{array}\right.$$

where *e* is the elementary charge, $\varepsilon_{0}$ is the vacuum permittivity, $\varepsilon_{r}=78.5$ is the relative permittivity of the solvent, $r_{ij}$ is the distance between the centers of particles *i* and *j*, $z_{i}$ is the charge number of *i*, and $\sigma_{ij}$ is the distance between the centers of particles *i* and *j* at contact. The Debye screening length ($\kappa^{-1}$) is calculated from the following equation:

(2) $$\kappa^{-1}=\sqrt{\frac{\varepsilon_{0}\varepsilon_{r}k_{B}T}{2\times 10^{3}N_{A}e^{2}I}}$$

where $k_{B}$ is Boltzmann’s constant, *T* is the temperature, $N_{A}$ is Avogadro’s constant and *I* is the ionic strength of the solution.

The radius of the amino acid spheres was set to 2.0 Å to allow for realistic distances between bonded residues. Bonded amino acids are connected with a harmonic potential:

(3) $$w_{b}=k_{h}{\left(R_{b}-R_{\mathrm{eq}}\right)}^{2}$$

where $k_{h}=0.76k_{B}T/$Å${}^{2}$ is the spring constant, $R_{b}$ is the bond length between the centers of the spheres, and $R_{\mathrm{eq}}=4.1$ Å is the equilibrium distance of the harmonic potential measured between the centers of the spheres. Non-bonded spheres also interact via a Lennard–Jones potential:

(4) $$w_{\mathrm{LJ}}=4\varepsilon_{\mathrm{nb}}\left[{\left(\frac{\sigma_{ij}}{r_{ij}}\right)}^{12}-{\left(\frac{\sigma_{ij}}{r_{ij}}\right)}^{6}\right]$$

where $\varepsilon_{\mathrm{nb}}=0.05k_{B}T$ is the interaction strength. The smooth surface was modeled as completely flat with a smeared charge distribution. The electrostatic interactions between the charged amino acids and the surface were modeled using Gouy–Chapman theory for a 1:1 salt:

(5) $$\begin{array}{cc} w_{\mathrm{GC}} & =2z_{i}k_{B}T\ln\left(\frac{1+\Gamma_{0}e^{-\kappa r_{s,i}}}{1-\Gamma_{0}e^{-\kappa r_{s,i}}}\right), \end{array}$$

(6) $$\begin{array}{cc} \Gamma_{0} & =\tanh\left[\frac{1}{2}\sinh^{-1}\left(\frac{\rho }{\sqrt{8k_{B}Tc_{0}\varepsilon_{0}\varepsilon_{r}}}\right)\right] \end{array}$$

where $r_{s,i}$ is the distance between the surface and the center of amino acid *i*, ρ is the surface charge density, and $c_{0}$ is the concentration of 1:1 salt. The concentration of 1:1 salt was set to 0.025 M.

The idea was to compare with the silica surfaces used in the experiments made by Hulander et al. [7], both with and without adsorbed silica nanoparticles. The surfaces were hydrophilic and therefore no hydrophobic attraction between the protein and the surface was included. The charges of the adsorbed nanoparticles were calculated to give the same surface charge density as that of the flat surface and the charges were placed in the centers of the spheres. Adsorption to free nanoparticles was also studied. The nanoparticles interacted with the amino acid beads according to Equation (1). The *z*-axis constituted the normal to the surface and the boundaries of the simulation box were periodic in the xy-directions. The side lengths of the simulation box varied between 150 and 420 nm depending on the system.

### 2.2. Method

The simulations were performed using the Metropolis Monte Carlo algorithm [31] in the NVT ensemble. The software used for the simulations was Faunus, a framework for Metropolis Monte Carlo simulations [32]. For the simulations of the main body of fibrinogen, two types of moves were used: translation and rotation of the fibrinogen entity. For the simulations of the αC-chains crankshaft moves (pick two beads randomly and define the vector connecting them as the rotation axis, and then rotate the beads between the picked ones around this axis), pivot moves (define a rotation axis in the same manner as with the crankshaft move, and then rotate the beads at one end of the protein around this axis) and single bead translation were also included.

### 2.3. Analysis

The angle between the protein main body and the surface of a nanoparticle was found in the following way: a line was drawn between the middle of the protein main body and the middle of the nanoparticle. Then, the cosine of the angle between this line and the protein was recorded.

The radius of gyration ($R_{g}$) of the αC-chain was found using the following equation:

(7) $$R_{g}^{2}=\frac{1}{M_{w}}\sum_{i=1}^{N}m_{i}\times {\left(r_{i}-r_{\mathrm{cm}}\right)}^{2},$$

where $M_{w}$ is the molecular weight of the αC-chain, $m_{i}$ is the mass of amino acid bead *i*, *N* is the number of beads, $r_{i}$ is the coordinates of bead *i*, and $r_{\mathrm{cm}}$ is the coordinates of the chain center-of-mass.

## 3. Results and Discussion

### 3.1. Characterization of Fibrinogen

**Figure 2:**
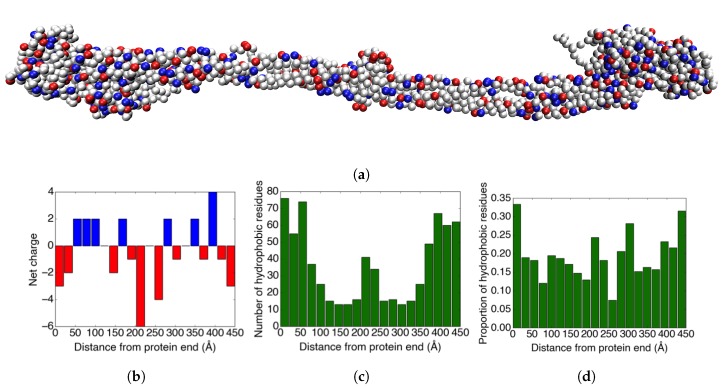
(**a**) An image of the coarse-grained fibrinogen body from the crystal structure. The red amino acid residues are negatively charged, the blue residues are positively charged, and grey represents neutral residues. (**b**) The charge distribution in the longitudinal direction of the fibrinogen crystal structure. (**c**) The distribution of hydrophobic amino acid residues in the longitudinal direction of the fibrinogen crystal structure. (**d**) The proportion of the ≥20% solvent-accessible amino acid residues that are hydrophobic for each bin.

The net charge of the main body of fibrinogen present in the crystal structure is −8 (counting also the bound calcium ions). When including the flexible parts of the Aα-chains that are not visible in the crystal structure, the net charge becomes −12. Figure 2b shows how the charges are distributed over twenty bins in the longitudinal direction of the fibrinogen crystal structure. While the ends and the middle of the protein are net negatively charged, there are also parts of the protein that are net positively charged. These are potentially important for the ability of fibrinogen to adsorb to negatively charged surfaces.

In addition to the charge distribution, the distribution of hydrophobic amino acids on the surface of the protein could also be important for adsorption. Therefore, we studied how the hydrophobic amino acid residues (Ala, Gly, Ile, Leu, Met, Phe, Pro, and Val) are distributed over the protein. Figure 2c shows how the hydrophobic amino acid residues are distributed over the crystal structure of fibrinogen. As expected, there is a clear correlation between the thickness of the protein and the number of hydrophobic amino acid residues (cf. Figure 2a).

Figure 2d displays the proportion of the surface-accessible residues that are hydrophobic for 20 bins in the longitudinal direction of the fibrinogen crystal structure. The solvent-accessible residues were found using Swiss PDB viewer [33], and the threshold for counting the residue as solvent-accessible was set to ≥20% solvent accessibility. The results show that the ends of the protein have a surface that is somewhat more hydrophobic than the rest of the protein.

### 3.2. Adsorption Simulations of the Main Body of Fibrinogen

In the simulations, we studied how different surface charges (density and sign), as well as the presence of charged spherical nanoparticles on the flat surface, affect adsorption of fibrinogen. We also compared adsorption onto nanoparticles with different size and different spacing on the flat surface. The aim was mainly to explain the finding that adsorbed fibrinogen increases the amount of *S. epidermidis* that adheres to a flat surface, while the presence of adsorbed fibrinogen has the opposite effect when nanoparticles are attached to the flat surface [7].

#### 3.2.1. Adsorption onto a Flat Surface

The surface charge densities used were ±0.001e /Å${}^{2}$, ±0.005e/Å${}^{2}$, and ±0.01e/Å${}^{2}$. Mainly the negatively charged surfaces were investigated. A realistic surface charge density for silica at pH 7.4 and ionic strength 0.025 M would be approximately −0.001e/Å${}^{2}$ according to experimental measurements [34,35]. However, according to simulation results, a realistic surface charge density is −0.005e/Å${}^{2}$ (see the Supplementary Materials).

**Figure 3:**
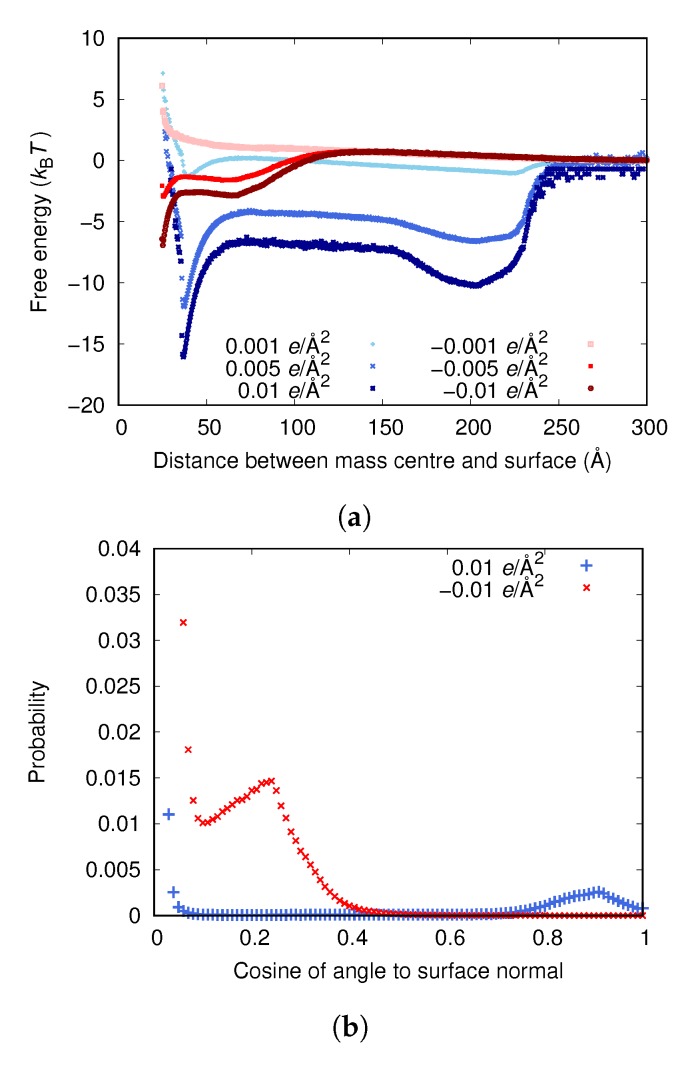

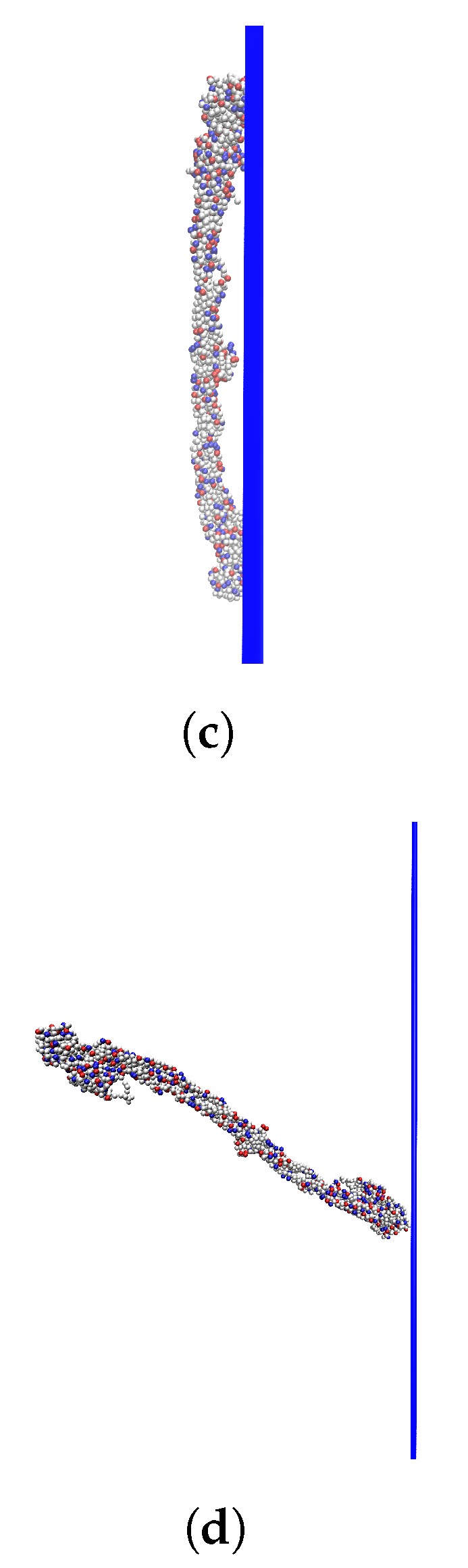
(**a**) The adsorption free energies for the fibrinogen main body to surfaces with different surface charge densities. (**b**) Distribution of cosine of the angle between the surface normal and an adsorbed protein for the higher surface charge densities. (The upper limit of the *y*-axis has been set so that it is possible to view peaks other than the ones for small cosine.) (**c**) A configuration with cosine close to 0, corresponding to the deepest free energy well when the surface charge is 0.01e/Å${}^{2}$. (**d**) A configuration with high cosine, corresponding to the second free energy well when the surface charge is 0.01e/Å${}^{2}$.

Figure 3a shows that, for both positive and negative surface charges, the fibrinogen body adsorbs solely due to electrostatic forces if the surface charge is high enough. It seems that different orientations of the protein are preferred depending on the sign of the surface charge, with the protein being more inclined to stand up at the surface if the charge is positive (the mass center of the protein is approximately half of a fibrinogen length away from the surface at one of the two free energy minima). This is also illustrated in Figure 3b, showing the distribution of the cosine of the angles between the adsorbed proteins and the surface normal for the highest surface charge densities. A value of 0 corresponds to the protein lying flat on the surface and a value of 1 to the protein standing up with its axis completely parallel to the normal of the surface. When the surface charge is positive, it is strongly favored that an adsorbed protein lies flat against the surface, although there is also a small maximum at a cosine of ∼0.9, which is completely absent when the surface charge is negative.

The fact that the fibrinogen main body adsorbs to both positively and negatively charged surfaces, if they are charged enough, emphasizes the importance of local electrostatic interactions between different charged groups in the protein and at the surface. However, since no adsorption is observed at the experimentally determined surface charge of approximately −0.001e/Å${}^{2}$ (see Figure 3), something is clearly missing in the model. It is probable that the experimental methods do not yield the surface charge exactly at the surface. Kubiak et al. calculated a surface charge density by using Gouy–Chapman theory to convert ζ-potential to charge density [24]. Even though the charge of the slipping plane (where the ζ-potential is measured) should probably be considerably lower than the surface charge, they found the same charge magnitude as those measured by Bolt [34] and Samoshina et al. [35]. Therefore, the surface charge of −0.005e/Å${}^{2}$ determined by simulations (see the Supplementary Materials) may be more reliable.

It might also be the case that other attractive forces need to be included in the simulations in order for them to match experiments. These could represent van der Waals forces or hydrogen bonding between the surface and the protein. In addition, electrostatic attraction tends to increase when having explicit surface charges and explicit ions in the solvent, which we did not have. Another possibility is that the repulsion is overestimated since the amino acids are treated as hard spheres.

#### 3.2.2. Adsorption Onto a Surface with Attached Nanoparticles

Table 1 shows a comparison between the adsorbed fractions of fibrinogen on the flat surfaces and the surfaces with attached nanoparticles. The nanoparticles had a radius of 20 nm and the surface coverage was 40%. The nanoparticles were hexagonally packed. The simulations show that fibrinogen adsorbs more often on the nanostructured surfaces than on the flat surfaces in the simulations, in agreement with previous QCM-D measurements [7].

The fact that adsorption is higher on nanostructured surfaces is partly due to the fact that they have a larger available surface area due to the added nanoparticles. However, Rechendorff et al., who studied adsorption of fibrinogen onto surfaces with different degrees of roughness, found that the adsorbed amount of fibrinogen increased more with roughness than the available surface area did [36]. Using Monte Carlo simulations, they found that fibrinogen is likely to adsorb to a higher degree with an end-on orientation on the rough surfaces, increasing the number of fibrinogen molecules that can bind.

Another reason for the increase in adsorption could be that the total charge of the nanoparticles and the flat surface is larger than that of the flat surface alone, which should increase the adsorption according to Figure 3a.

**Figure 4:**
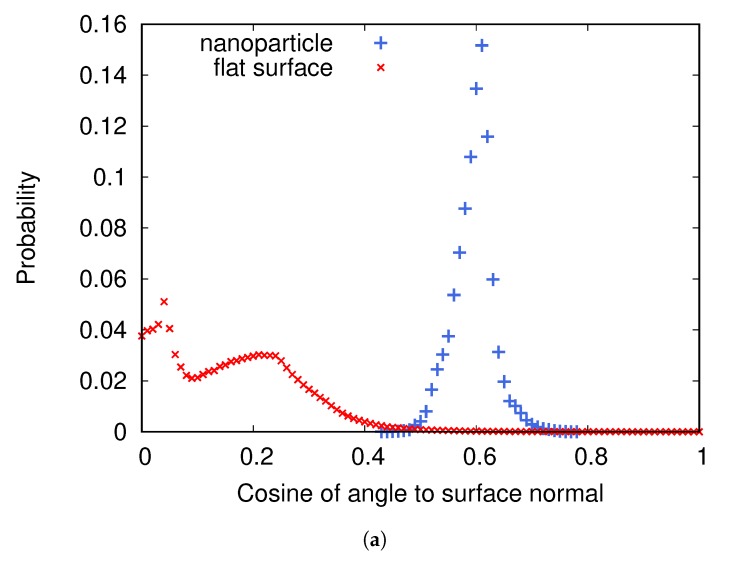

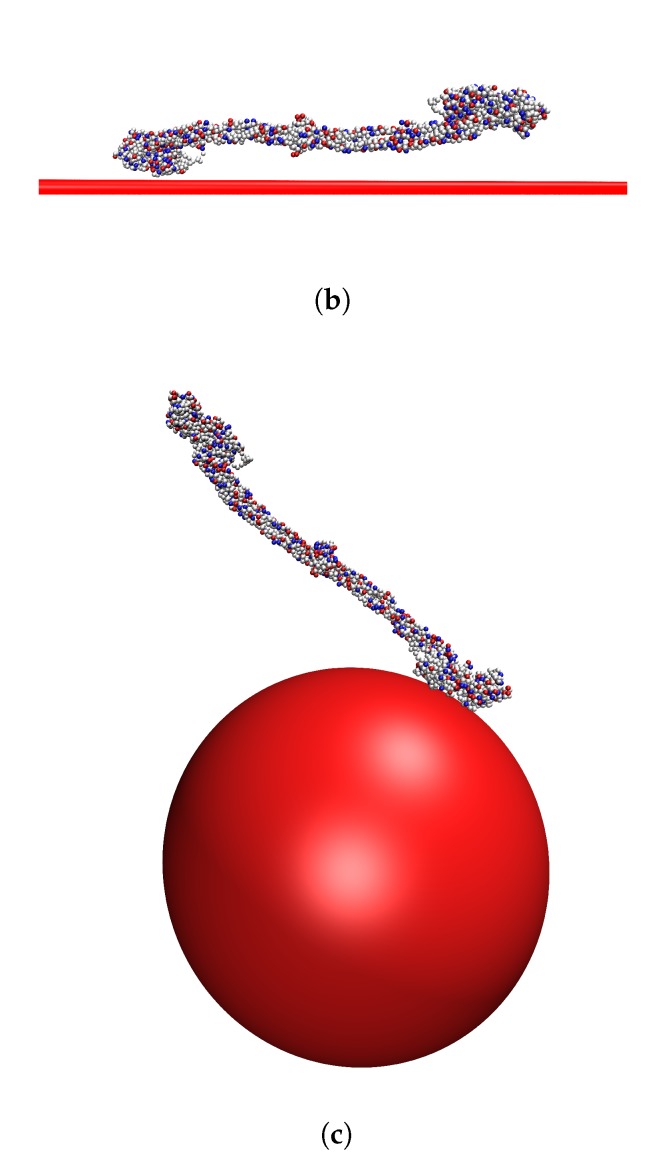
(**a**) The distributions of cosine of the angle between fibrinogen and the normal to the surface it is adsorbed to for two cases: when adsorbed to a nanoparticle attached to a flat surface and when adsorbed to a flat surface without nanoparticles. The surface charge density is −0.005e/Å${}^{2}$. (**b**) A typical orientation of fibrinogen adsorbed on the flat surface. (**c**) A typical orientation of fibrinogen adsorbed on a nanoparticle.

Figure 4a shows the distributions of cosine of the angle between a nanoparticle and an adsorbed fibrinogen and between the flat surface and an adsorbed fibrinogen when the surface charge density is −0.005e/Å${}^{2}$. On the flat surface, fibrinogen prefers to adsorb with the whole main body close to the surface, while on the nanoparticles it protrudes further out in the solution. Figure 4b,c shows two preferred orientations of fibrinogen adsorbed on the two types of surfaces.

It appears that one of the D domains adsorbs close to the surface, and that the angle between the D domain and the rest of the fibrinogen rod, combined with the surface curvature, determines the angle between the entire fibrinogen main body and the surface.

#### 3.2.3. Adsorption Onto Nanoparticles with Different Size

**Figure 5:**
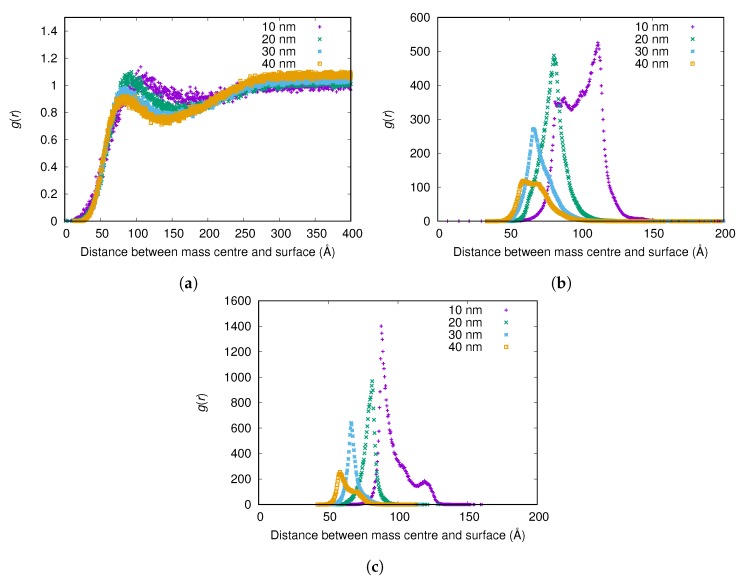
The radial distribution function *g*(*r*) between the surface of the nanoparticle and the mass center of the main body of fibrinogen for different nanoparticle radii and surface charge densities: (**a**) $-0.001e/{Å}^{2}$; (**b**) $-0.005e/{Å}^{2}$; and (**c**) $-0.01e/{Å}^{2}$. The *g*(*r*) are normalized using the (constant) concentration of fibrinogen in the simulation box instead of a bulk concentration for each simulation, since reliable bulk concentrations could not be determined from the simulations with highest attraction to the surface.

The results show that fibrinogen adsorbs more strongly to small nanoparticles (see Figure 5), and more fibrinogen adsorbs to smaller nanoparticles per surface area (see Table 2). (Since essentially all protein adsorbs for the higher surface charges, only results from the lowest charge are shown here and in corresponding tables). However, while more fibrinogen adsorbs to small nanoparticles than larger ones per surface area, less fibrinogen adsorbs to the small nanoparticles than the larger ones if comparing adsorbed amount per nanoparticle (see Table 2).

The fact that less fibrinogen adsorbs to the small nanoparticles than the larger ones, if comparing the adsorbed amount per nanoparticle, is of course due to the larger surface area of the larger nanoparticles. This result is in agreement with the results of Lundqvist et al., showing a larger protein adsorption on larger nanoparticles for a constant nanoparticle concentration [37]. However, Lundqvist et al. could not determine whether this was due to the difference in curvature of the particles or the difference in available surface area between the samples.

The explanation for the observation that fibrinogen binds more strongly to smaller nanoparticles may lie in the fact that the ends of the rigid part of the fibrinogen molecule are approximately neutral (see Figure 2b), with several positive charges available for favorable interaction with the surface, while the middle of the molecule is negatively charged. Thus, if the positive charges at one end of the protein bind to the negative surface in the same way regardless of the surface curvature, the adsorption is more favorable the further away from the surface that the negatively charged middle part of the protein ends up.

**Figure 6:**
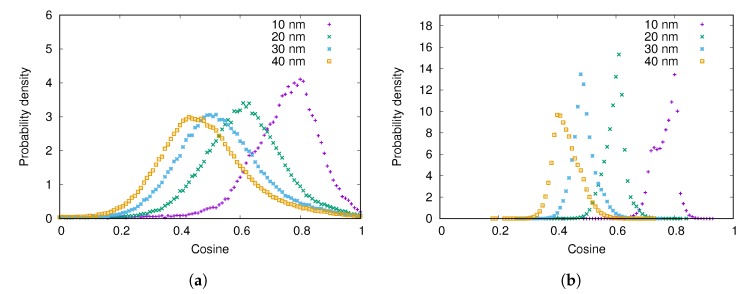
The distribution of cosine of the angle between the surface normal and the adsorbed fibrinogen main body for different nanoparticle radii when the surface charge density is: (**a**) $-0.001e/{Å}^{2}$; (**b**) $-0.005e/{Å}^{2}$.

The angle between the adsorbed fibrinogen molecule and the surface of the nanoparticle increases as the size of the nanoparticle decreases (see Figure 6). This is due to the fact that fibrinogen adsorbs with one of its ends on the surface of the particle in a similar way regardless of the nanoparticle size, and the higher is the curvature of the particle, the larger is the angle. Similar results were obtained by Lopez and Lobaskin for fibrinogen adsorption onto hydrophobic nanoparticles [20].

#### 3.2.4. Adsorption Onto Nanostructured Surfaces with Different Nanoparticle Size

Here, adsorption of the fibrinogen main body onto surfaces with attached nanoparticles of different sizes are compared. Firstly, the number density of nanoparticles on the surface is held constant (distance between centers of nearest neighbors = 60 nm) and, secondly, the surface coverage of the nanoparticles is constant (40%). The nanoparticle radii are 10, 20, and 30 nm. On all surfaces studied here, the nanoparticles were packed hexagonally. When the nanoparticle number density is constant, the adsorbed fraction of fibrinogen is highest for a nanoparticle radius of 20 nm (see the second column of Table 3). When the nanoparticle radius is increased from 10 to 20 nm, the available surface area increases since the nanoparticles become larger and thus contribute with more surface area. However, when the nanoparticle radius is further increased to 30 nm, the spheres are in contact with each other and thus the available surface area decreases. Thus, in this case, the nanoparticles with 20 nm radius give maximum adsorption since they give the largest available surface area.

When the nanoparticle surface coverage is constant at 40%, the adsorbed fraction increases with nanoparticle size for all sizes used, see the third column of Table 3. Here, the total surface area of the spheres is the same for all three nanoparticle radii. Thus, it could be expected from the results of the simulations with single nanoparticles that more fibrinogen would adsorb onto the surface with small nanoparticles. However, when the small nanoparticles are attached to a flat surface, a larger proportion of the surface area of each sphere is blocked by the flat surface than for the larger spheres. This makes the surface area that is available for fibrinogen adsorption smaller for surfaces with smaller attached nanoparticles.

#### 3.2.5. Adsorption Onto Nanostructured Surfaces with Different Nanoparticle Spacing

**Figure 7:**
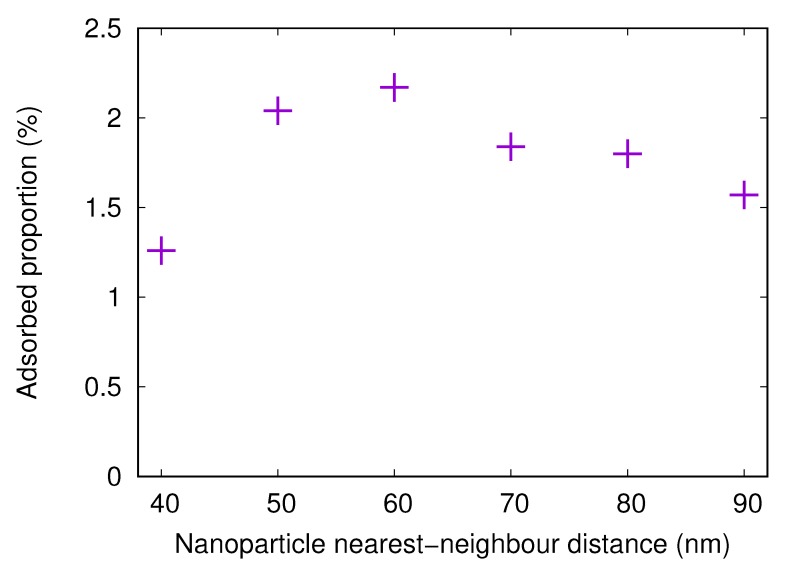
The adsorbed proportion of the fibrinogen main body in simulations with flat surfaces with attached nanoparticles (20 nm radius) with different distances between centers of nearest neighbors. The surface charge density for the flat surface and the nanoparticles is −0.001e/Å${}^{2}$.

Simulations with nanostructured surfaces with nanoparticles of 20 nm radius were performed for different spacings between the nanoparticles. The center-to-center distance between nearest neighbors was varied between 40 and 90 nm in steps of 10 nm. The adsorbed fractions are shown in Figure 7.

The adsorbed amount onto a surface with attached nanoparticles with a radius of 20 nm increases when the nearest neighbor center-to-center distance increases from 40 to 60 nm. This is due to the fact that, when the nanoparticles are close to each other, it is difficult or impossible for the protein to adsorb between the nanoparticles. However, for 60 nm spacing, the available surface area is large. As the spacing increases even more, the nanoparticles become fewer, thus reducing the available surface area and the adsorbed amount.

### 3.3. Simulations of the αC-Chain of Fibrinogen

#### 3.3.1. Simulations in Bulk and with a Flat Surface

**Figure 8:**
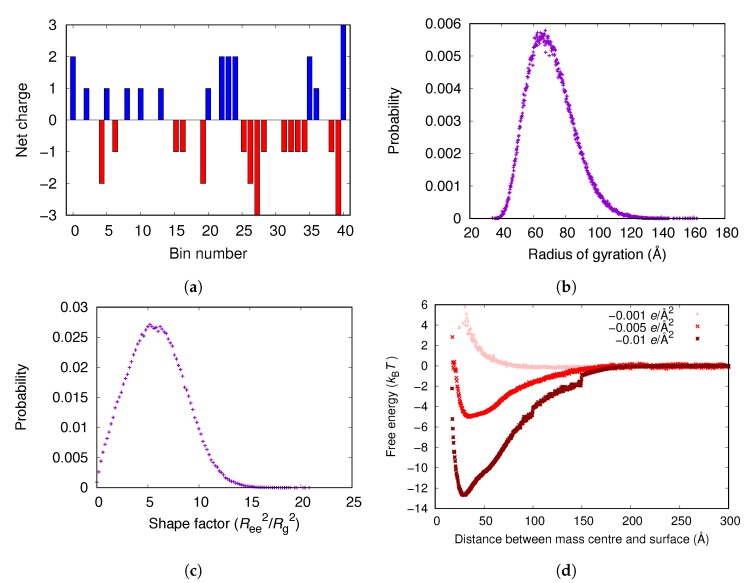
(**a**) The distribution of charges of the αC-chain divided into 41 bins (10 amino acid residues per bin) starting from the N-terminal. The negative charge of the C-terminal is included. (**b**) The distribution of the radius of gyration of the αC-chain in bulk. (**c**) The distribution of the shape factor of the αC-chain in bulk. (**d**) The adsorption free energies for the αC-chain to surfaces with different negative surface charge densities.

The charges of the αC-chain are relatively evenly distributed over the molecule (see Figure 8a). However, some parts of the chain are net positively charged, possibly contributing to the adsorption of the chain on a negatively charged surface. Figure 8d shows that the αC-chain adsorbs electrostatically to surfaces with charge densities of −0.005 e/Å${}^{2}$ and −0.01 e/Å${}^{2}$, as the main body of fibrinogen did. However, the free energy minima for the αC-chain is lower than for the fibrinogen main body (Figure 3a). Figure 8b shows the distribution of the radius of gyration of the αC-chain in bulk and Figure 8c shows the distribution of the shape factor, $R_{\mathrm{ee}}^{2}/R_{g}^{2}$, where $R_{\mathrm{ee}}$ is the end-to-end distance and $R_{g}$ is the radius of gyration. A shape factor of 6 is characteristic of a Gaussian chain and a shape factor of 12 corresponds to a rigid rod. Thus, the αC-chain behaves as a Gaussian chain in the simulations.

A comparison between the free energies in Figure 3 and Figure 8 shows that the αC-chains are important for the adsorption of the complete fibrinogen since the free energy minimum is lower than for the fibrinogen main body.

#### 3.3.2. Adsorption Onto Different Types of Surfaces

Table 4 shows the proportions of the αC-chain that adsorbs to a flat and a nanostructured surface, respectively, in the simulations. The nanostructured surface is the same as in Section 3.2.2. The trends are the same as for the main body of fibrinogen (Table 1) but the adsorbed amounts of the αC-chain are higher, again showing the importance of the αC-chains for the overall adsorption.

#### 3.3.3. Adsorption Onto Nanoparticles with Different Size

**Figure 9:**
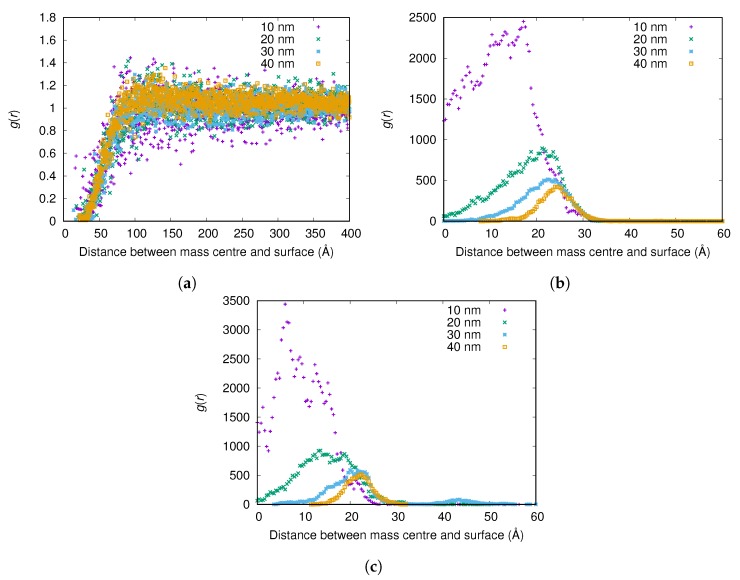
The radial distribution function *g*(*r*) between the surface of the nanoparticle and the mass center of the αC-chain for different nanoparticle radii and surface charge densities: (**a**) −0.001e/Å${}^{2}$; (**b**) −0.005e/Å${}^{2}$; and (**c**) −0.01e/Å${}^{2}$. The *g*(*r*) are normalized using the (constant) concentration of the αC-chain in the simulation box instead of a bulk concentration for each simulation, since reliable bulk concentrations could not be determined from the simulations with highest attraction to the surface.

From g(r) in Figure 9a, no significant difference can be observed between the adsorption of the αC-chain of fibrinogen onto nanoparticles with radii of 10, 20, 30, and 40 nm when the surface charge density is −0.001e/Å${}^{2}$. However, when the surface charge density is −0.01e/Å${}^{2}$, the αC-chain adsorbs more strongly to the smallest nanoparticle (radius 10 nm) (see Figure 9b).

Table 5, however, shows that more of the αC-chain adsorbs on the smaller nanoparticles (radii 10 and 20 nm) per surface area also for the lower surface charge density of −0.001e/Å${}^{2}$.

The trends here are the same as for the main body of fibrinogen. The reason could be that, due to the smaller surface area of the 10-nm nanoparticles, negative amino acid residues on an adsorbed protein that are further away from the surface do not feel as much repulsion as they would in the same conformation adsorbed on a larger nanoparticle.

#### 3.3.4. Adsorption Onto Nanostructured Surfaces with Different Nanoparticle Size

Here, the results from adsorption simulations of the αC-chain onto the same nanostructured surfaces as studied for the fibrinogen main body (Section 3.2.4) is presented. The trends for the adsorbed fractions are the same as for the fibrinogen main body (cf. Table 3 and Table 6). The reason is the same as described previously—the trends follow the available surface area.

#### 3.3.5. Adsorption Onto Nanostructured Surfaces with Different Nanoparticle Spacing

**Figure 10:**
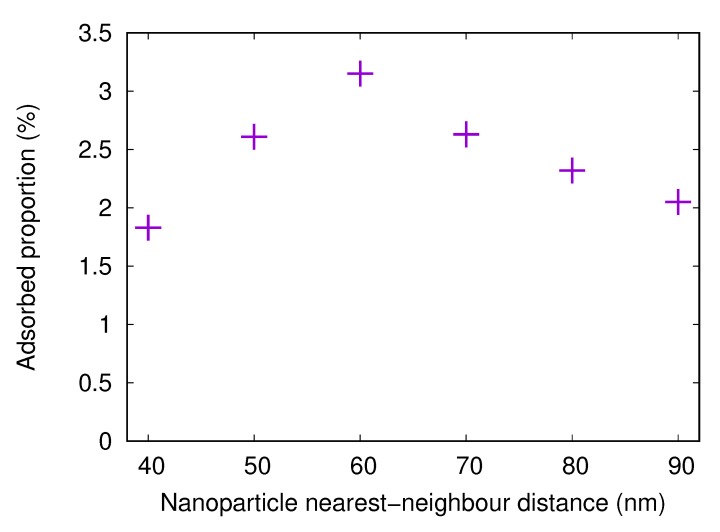
The adsorbed proportion of the fibrinogen αC-chain in simulations with flat surfaces with attached nanoparticles (20 nm radius) with different distances between centers of nearest neighbors. The surface charge density for the flat surface and the nanoparticles is −0.001e/Å${}^{2}$.

Here, results from simulations with nanostructured surfaces with nanoparticles of 20 nm radius and different spacings are presented. The adsorbed fractions are shown in Figure 10. The trends are the same as for the fibrinogen main body, with the highest adsorbed amount when the distance between the centers of neighboring particles is 60 nm. Here, the optimal combination of available surface area *between* the particles and available surface area *on* the particles is found.

## 4. Conclusions

We used coarse-grained simulations to study the adsorption of the fibrinogen main body, as well as the fibrinogen αC-chain, on negatively charged smooth surfaces, nanospheres, and flat surfaces with attached nanospheres.

Our study confirms that the disordered αC-chains of fibrinogen are important for adsorption on negatively charged surfaces. A single αC-chain attaches more firmly than the fibrinogen main body.

When fibrinogen adsorbs to surfaces with attached nanospheres, the available surface area is generally what determines the proportion of fibrinogen that is adsorbed. However, when the different parts of the fibrinogen molecule are adsorbed to spherical nanoparticles in solution, the adsorbed amount per surface area increases as the size of the nanoparticle decreases. The reason could be that that negatively charged amino acid residues feel less repulsion from the negatively charged nanoparticle the smaller it is, since the surface area is smaller.

We found that the main body of fibrinogen protrudes more into solution as the curvature of the surface increases, and that the main body of fibrinogen is anchored to the surface through one of is D-domains. This was found true regardless of the surface curvature and the orientation therefore seems to be solely a function of the surface curvature. Thus, we hypothesize that fibrinogen adsorbed on surfaces with attached nanoparticles makes cell-binding epitopes less available to attaching cells. This could explain previous findings where the adhesion of *S. epidermidis* was hampered on fibrinogen adsorbed to nanostructured compared to smooth substrates.

## Figures and Tables

**Table 1:** The proportions of the fibrinogen main body that are adsorbed in simulations with flat and nanostructured surfaces.

Surface Type	Charge Density (*e*/Å${}^{2}$)	Adsorbed Proportion (%)
Flat	−0.001	0.503
Nanostructured	−0.001	2.17
Flat	−0.005	7.88
Nanostructured	−0.005	100
Flat	−0.01	39.2
Nanostructured	−0.01	100

**Table 2:** The adsorbed proportion of the fibrinogen main body per surface area in simulations with a single nanoparticle with different radii when the surface charge density is −0.001e/Å${}^{2}$.

Nanoparticle Radius (nm)	Adsorbed Proportion (%)	Adsorbed Proportion/Surface Area ($10^{-6}$ nm${}^{-2}$)
10	0.179	3.78
20	0.474	1.64
30	0.825	0.729
40	1.27	0.628

**Table 3:** The adsorbed proportion of the fibrinogen main body in simulations with flat surfaces with attached nanoparticles with different radii for a fixed distance between centers of nearest neighbors (nn) and a fixed surface coverage of nanoparticles (sc). The surface charge density for the flat surface and the nanoparticles is −0.001e/Å${}^{2}$.

Nanoparticle Radius (nm)	Adsorbed Proportion (%), nn = 60 nm	Adsorbed Proportion (%), sc = 40%
10	1.05	1.50
20	2.17	2.17
30	1.46	2.73

**Table 4:** The proportions of the fibrinogen αC-chain that are adsorbed in simulations with flat and nanostructured surfaces.

Surface Type	Charge Density (*e*/Å${}^{2}$)	Adsorbed Proportion (%)
Flat	−0.001	1.23
Nanostructured	−0.001	3.14
Flat	−0.005	60.1
Nanostructured	−0.005	100
Flat	−0.01	100
Nanostructured	−0.01	100

**Table 5:** The adsorbed proportion of the αC-chain of fibrinogen per surface area in simulations with a single nanoparticle with different radii when the surface charge density is −0.001e/Å${}^{2}$.

Nanoparticle Radius (nm)	Adsorbed Proportion (%)	Adsorbed Proportion/Surface Area ($10^{-6}$ nm${}^{-2}$)
10	0.128	1.02
20	0.392	0.781
30	0.774	0.684
40	1.38	0.687

**Table 6:** The adsorbed proportion of the fibrinogen αC-chain in simulations with flat surfaces with attached nanoparticles with different radii for a fixed distance between centers of nearest neighbors (nn) and a fixed surface coverage of nanoparticles (sc). The surface charge density for the flat surface and the nanoparticles is −0.001e/Å${}^{2}$.

Nanoparticle Radius (nm)	Adsorbed Proportion (%), nn = 60 nm	Adsorbed Proportion (%), sc = 40%
10	1.54	1.69
20	3.15	3.15
30	1.95	3.34

## Supplementary Materials

The following are available online at https://www.mdpi.com/2218-273X/10/3/413/s1.
