# Supplementary material for: Adsorption of Fibrinogen on Silica Surfaces—The Effect of Attached Nanoparticles
Source: Biomolecules. 2020 Mar 6;10(3):413. doi: 10.3390/biom10030413 (PMC7175343; doi:10.3390/biom10030413)
Supplement: Supplementary file 1 [file biomolecules-10-00413-s001.pdf]

# Supplementary Material

Kristin Hyltegren, Mats Hulander, Martin Andersson, Marie Skepö

Table 1 shows the different sets of parameters from different sources that where used for simulating titrating silica surfaces with Faunus [6]. Tables 2–3 show the simulation results for the surface charge density and the ratio between simulated charge and the experimentally reported charge by Samoshina et al. [5] for ionic strengths of 10 and 100 mM, respectively. The charges were placed on a square lattice in the simulations.

On average, the surface charge density from simulations was 5.0 times the experimentally determined charge.

| **Parameter set** | **$pK_a$ 1** | **Percentage (%)** | **$pK_a$ 2** | **Percentage (%)** | **Silanol group density (Å$^{-2}$)** |
|---|---|---|---|---|---|
| 1 [4] | 7.6 | 100 | - | - | 0.08 |
| 2 [3] | 4.5 | 19 | 8.5 | 81 | 0.049 |
| 3 [2] | 5.5 | 15 | 9.0 | 85 | 0.049 |
| 4 [1] | 7.1 | 100 | - | - | 0.049 |

Table 1: The four sets of parameters that were used for titrating surface simulations and the references for the parameter sets. Some find that there are two types of silanol groups with different $pK_a$ values while others find only one type of group.

| **Parameter set** | **Charge density ($e$/Å$^2$)** | **Simulated charge/experimental** |
|---|---|---|
| 1 | -0.00198 | 3.17 |
| 2 | -0.00477 | 7.64 |
| 3 | -0.00318 | 5.09 |
| 4 | -0.00238 | 3.81 |

Table 2: Charge densities found from titrating silica surface simulations at pH 7 and ionic strength 10 mM and their relation to the experimentally determined surface charge.

| **Parameter set** | **Charge density ($e$/Å$^2$)** | **Simulated charge/experimental** |
|---|---|---|
| 1 | -0.00467 | 3.74 |
| 2 | -0.00877 | 7.03 |
| 3 | -0.00600 | 4.81 |
| 4 | -0.00595 | 4.76 |

## References

[1] Michael L Hair and William Hertl. “Acidity of surface hydroxyl groups”. In: *J. Phys. Chem.* 74.1 (1970), pp. 91–94.

[2] Lawrence H Allen, Egon Matijevıc, and Louis Meites. “Exchange of Na+ for the silanolic protons of silica”. In: *J. Inorg. Nucl. Chem.* 33.5 (1971), pp. 1293–1299.

[3] Shaowei Ong, Xiaolin Zhao, and Kenneth B Eisenthal. “Polarization of water molecules at a charged interface: second harmonic studies of the silica/water interface”. In: *Chem. Phys. Lett.* 191.3-4 (1992), pp. 327–335.

[4] Motoyoshi Kobayashi et al. “Aggregation and charging of colloidal silica particles: effect of particle size”. In: *Langmuir* 21.13 (2005), pp. 5761–5769.

[5] Yulia Samoshina et al. “Equilibrium Aspects of Polycation Adsorption on Silica Surface: How the Adsorbed Layer Responds to Changes in Bulk Solution”. In: *Langmuir* 21.13 (2005), pp. 5872–5881.

[6] Björn Stenqvist et al. “Faunus — a flexible framework for Monte Carlo simulation”. In: *Mol. Simul.* 39.14-15 (2013), pp. 1233–1239.
